# Fish consumption pattern among adults of different ethnics in Peninsular Malaysia

**DOI:** 10.3402/fnr.v60.32697

**Published:** 2016-08-16

**Authors:** Nurul Izzah Ahmad, Wan Rozita Wan Mahiyuddin, Tengku Rozaina Tengku Mohamad, Cheong Yoon Ling, Siti Fatimah Daud, Nasriyah Che Hussein, Nor Aini Abdullah, Rafiza Shaharudin, Lokman Hakim Sulaiman

**Affiliations:** 1 Institute for Medical Research, Kuala Lumpur, Malaysia; 2 School of Food Science and Technology, Universiti Malaysia Terengganu, Kuala Terengganu, Malaysia; 3 Ministry of Health Malaysia, Federal Government Administration Centre, Putrajaya, Malaysia

**Keywords:** seafood, fish, consumption pattern, Malaysian, ethnicity

## Abstract

**Background:**

Understanding different patterns of fish consumption is an important component for risk assessment of contaminants in fish. A few studies on food consumption had been conducted in Malaysia, but none of them focused specifically on fish consumption. The objectives of this study were to document the meal pattern among three major ethnics in Malaysia with respect to fish/seafood consumption, identify most frequently consumed fish and cooking method, and examine the influence of demographic factors on pattern of fish consumption among study subjects.

**Methods:**

A cross-sectional survey was conducted between February 2008 and May 2009 to investigate patterns of fish consumption among Malaysian adults in Peninsular Malaysia. Adults aged 18 years and above were randomly selected and fish consumption data were collected using a 3-day prospective food diary.

**Results:**

A total of 2,675 subjects, comprising male (44.2%) and female (55.7%) participants from major ethnics (Malays, 76.9%; Chinese, 14.7%; Indians, 8.3%) with a mean age of 43.4±16.2 years, were involved in this study. The results revealed 10 most frequently consumed marine fish in descending order: Indian mackerel, anchovy, yellowtail and yellow-stripe scads, tuna, sardines, torpedo scad, Indian and short-fin scads, pomfret, red snapper, and king mackerel. Prawn and squid were also among the most preferred seafood by study subjects. The most frequently consumed freshwater fish were freshwater catfish and snakehead. The most preferred cooking style by Malaysians was deep-fried fish, followed by fish cooked in thick and/or thin chili gravy, fish curry, and fish cooked with coconut milk mixed with other spices and flavorings. Overall, Malaysians consumed 168 g/day fish, with Malay ethnics’ (175±143 g/day) consumption of fish significantly (*p*<0.001) higher compared with the other two ethnic groups (Chinese=152±133 g/day, Indians=136±141 g/day).

**Conclusion:**

Fish consumption was significantly associated with ethnicity, age, marital status, residential area, and years of education of adults in Peninsular Malaysia, and the data collected are beneficial for the purpose of health risk assessment on the intake of contaminants through fish/seafood consumption.

Populations in eastern Asia consumed fish with cooked rice daily, or as part of rice dishes or as side dishes (1). Malaysians, in particular, consumed fish at least once a day in the amounts of one and one-half medium fish per day (2). In fact, the annual per capita fish consumption of Malaysian was the second highest after Japan, among Asian nations, or ranked number fifth throughout the world (3). The consumption of fish is an essential part of a healthy and well-balanced diet (4). Potential health benefits related to fish consumption are due to the presence of protein, unsaturated essential fatty acids, minerals, and vitamins (5). Additional health benefits from the consumption of fish or fish oil may relate to polyunsaturated fatty acids (PUFAs), especially eicosapentaenoic acid (EPA) and docosahexaenoic acid (DHA) (5, 6). Fish provide omega-3 fatty acids that could reduce cholesterol levels and the incidence of heart disease, stroke, and preterm delivery (7). It also acts as a mood stabilizer (8), especially among females (9).

In contrast to the potential health benefits of dietary fish intake, fish could be contaminated with environmental toxicants that may pose health risk to human (6, 10, 11). Major chemical contaminants in fish were methyl mercury and polychlorinated biphenyls, while other potential toxic contaminants were polychlorinated dibenzo-*p*-dioxins, dibenzofurans, and polycyclic aromatic hydrocarbons or pollutants such as polybrominated diphenyl ethers, polychlorinated diphenyl ethers, and polychlorinated naphthalenes (PCNs) (6, 12, 13). These contaminants were the highest contributors to the human daily intake through fish consumption (6). Other possible hazard contributors were cadmium and hexachlorobenzene (6, 12, 14).

Understanding the patterns of fish consumption is the key factor for assessing exposure to harmful chemicals from the ingestion of contaminated fish. Fish consumption is one item that forms the basis of food consumption patterns that are defined as the consumption of specific food items and their combination in dishes and meals (15). Many factors affect fish consumption patterns, which vary widely between countries, and among different cultures as a result of the availability of commodities and economic factors (3, 4, 15–18). The most prominent measures of food consumption behavior are income and price elasticity (19). Other than evaluating the intake and exposure of various contaminants, fish consumption pattern is also important for exploring the difference in patterns, assessing the adequacy of nutrient intake, plus establishing policies in agriculture, production, trade, and health (2, 16, 19).

The objectives of this study were to document the meal patterns among three major ethnics in Malaysia with respect to fish/seafood consumption, and identify the most frequently consumed fish and cooking method. It is also aimed to examine the influence of demographic factors on the pattern of fish consumption among study subjects. The results of this study can be used as a baseline for estimating and assessing the risk of contaminated the seafood consumed by Malaysians.

## Material and methods

### Study design and subjects

A house-to-house survey was conducted and data were collected through face-to-face interviews using pre-design questionnaires in Peninsular Malaysia, between February 2008 and May 2009. The sampling frame used for the selection of study subjects’ household addresses was based on the National Household Sampling Frame (NHSF), Department of Statistics, Malaysia (20). This sampling frame was made up of enumeration blocks (EBs) created for the 2,000 Population and Housing Census. EBs are geographical contiguous areas of land with identifiable boundaries. On average, each EB contains about 80 to 120 living quarters. Generally, all EBs were formed within gazetted boundaries, particularly administrative districts, mukim, or local authority areas.

In order to obtain a representative sample, a two-stage proportionate stratified sampling technique was applied with states as the primary sampling units and urban or rural status as the secondary sampling units. Interviews were done with the head of the household or adult males and females aged 18 years and above residing in the house. The only exclusion criterion was pregnant women.

The sample size (*N*) calculation was as below:

$$\text{Formulation }N=(Z_{\alpha }^{2}\text{P}(1-\text{P}))/{\text{E}}^{2}\approx \text{208}\times \text{factors of different areas},\text{ethnicity},\text{and age}$$

where:

*Z* = 1.96 (based on 95% CI)

*P* = Prevalence of seafood consumption in grams per person per day. Based on consumption survey data for the Selangor population (16.2%)

*E* = Maximum tolerance error (5%)

α = 0.05 at 95% CI

Sample size calculated includes 20% expected drop – out

The calculation was based on consumption survey data for the Selangor population, in which the adult population of Selangor consumed fish at 16.2% (153 g/person/day compared with 944 g/person/day total food) (21). In addition, factors of the two different areas (urban and rural), three major ethnics (Malays, Chinese, and Indian), and two different age groups were used at the final stage. The number, 2,496 subjects, was required in order to obtain 95% confidence interval and a 5% margin of error. Taking into account a 20% dropped-off rate, 2,996 subjects were identified. A minimum count of two adults in each household was selected in this survey, and 1,500 household addresses were identified from the NHSF.

**Fig. 1:**
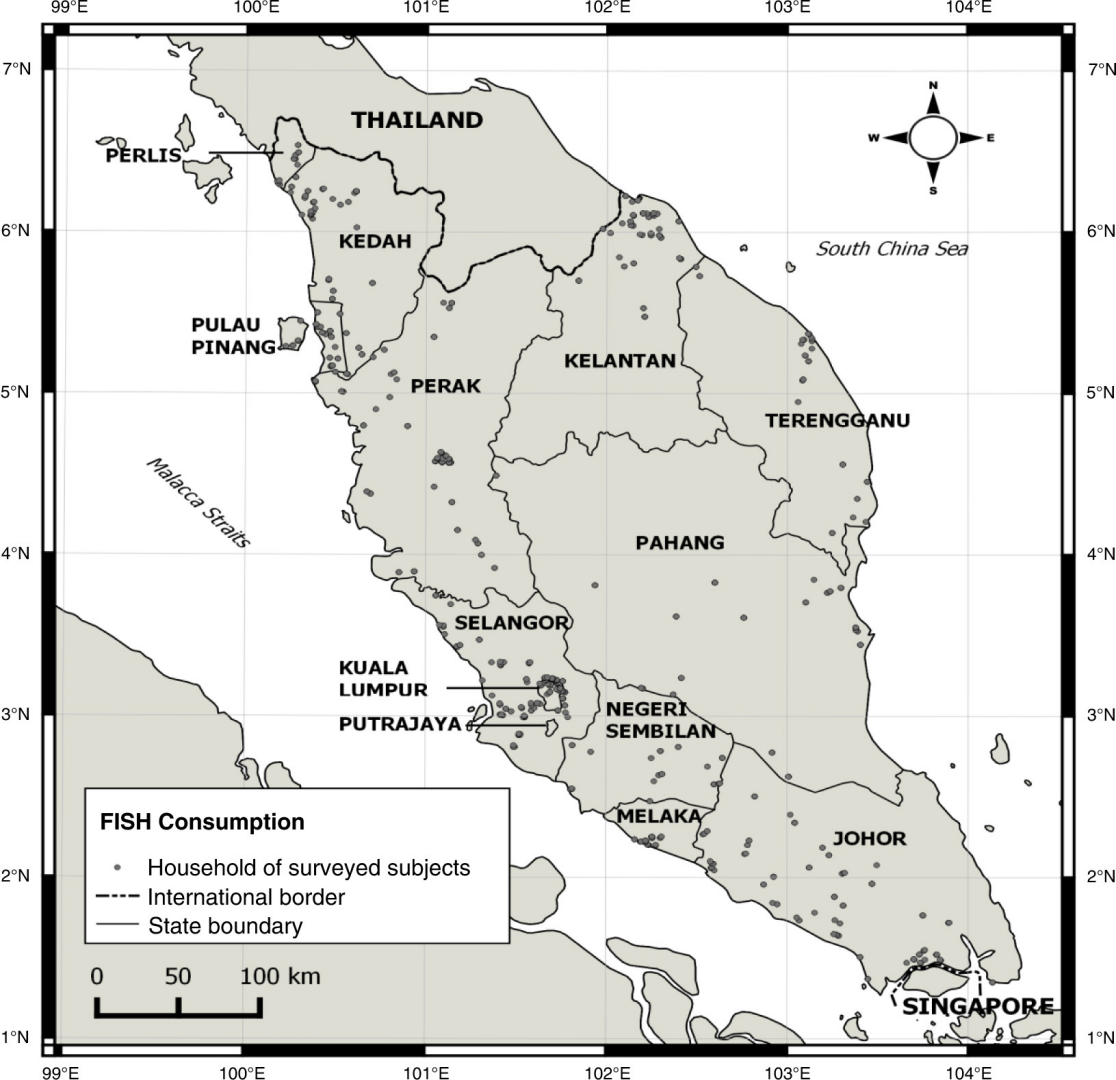
The household addresses of study subjects plotted throughout Peninsular Malaysia using Quantum GIS 2.8.1.

At the end of the survey, only 2,704 participated, and a final count of 2,675 subjects completed the questionnaire. Figure 1 showed the household addresses of study subjects plotted throughout Peninsular Malaysia using Quantum GIS 2.8.1. The response rate was 89.2%, and the sample characteristics are given in Table 1.

**Table 1:** Socio-demographic characteristics of the subjects

Socio-demographic Variables	Malaysian (*n*=2,058)	Chinese (*n*=394)	Indian (*n*=223)	Overall Total (*n*=2,675)	*p
Age (year±SD)	42.7±16.2^a^	48.1±16.3^b^	41.5±15.1^a^	43.4±16.2	0.000
Gender (%)					
Male	44.5	45.9	37.4	44.2	–
Female	55.4	54.1	62.6	55.7	
Body weight (kg±IQR)	62.0±17.9	60.5±16.8	63.9±20.0	62.0±17.5	**0.001
Height (cm±SD)	158.0±8.7^a^	160.2±9.1^b^	160.3±9.5^b^	158.5±8.8	0.000
BMI (±SD)	25.6±5.5^a^	24.4±4.9^b^	26.4±6.1^c^	25.4±5.5	0.000
Household number (±SD)	5.2±2.3^a^	4.6±2.4^b^	4.9±2.1^c^	5.1±2.3	0.000
Marital status (%)					
Single	21.1	19.1	19.5	20.6	
Married	71.5	73.4	71.4	71.9	–
Widowed/divorced	7.4	7.5	9.1	7.5	
Residential region (%)					
North	30.4	36.9	50.7	33.0	
Middle	25.1	17.0	39.5	25.2	
South	16.0	36.6	6.3	18.2	
East	28.6	9.4	3.6	23.7	–
Years of education (year±SD)	9.1±3.9^a^	8.4±4.1^b^	9.1±3.7^a^	9.0±3.9	0.004

IQR is the interquartile range. SD is the standard deviation.

* Significant difference (*p*<0.05) between different ethnics was evaluated using a one-way ANOVA. Equal variance was assumed using LSD.

** ^KW^ A Kruskal–Wallis U test was applied (χ^2^=13.956).

Different alphabets within the same row indicate significant difference (*p*<0.05).

### Questionnaire

The study instrument used was a set of questionnaires that had been validated prior to the study by distributing the questionnaires to other researchers who were not involved in the study. The questionnaires consisted of two parts. The first part comprised nine pages of self-administered questions, which consisted of a socio-demographic information section as well as questions on the pattern of fish consumption, frequency of fish consumption, and a final section on knowledge, perception, and practices toward fish consumption. The second part was three copies of 24-h dietary diary forms. In this part, subjects were asked to record food and drinks they consumed at every meal of the day. The form was divided into six meal sections (breakfast, morning tea, lunch, afternoon tea, dinner, and supper) that required recording the time, place, and with whom the subjects took each meal. They were also required to record types of food and drinks, the portion size, and cooking style.

The interviewers were trained to review and understand the questionnaires. During training, they were taught how to give instructions to subjects. They were equipped with a set of questionnaire tools to help the subjects record the type of foods they consumed. The questionnaire tools included pictures of serving dishes; fish commonly found in Malaysia; and common household measures, such as standard measuring cups, bowls, ladles, and spoons. The self-administrated questionnaire was given between 9.00 a.m. and 6.00 p.m. but sometimes interviewers had to visit at night because subjects were not home during the day. Interviewers assisted by reading the questionnaires to some of the elderly or illiterate respondents. They also rechecked all food recorded in dietary diary forms to verify the types and amounts of food consumed by subjects.

The portion weight of food was referred to the local food atlas ‘Atlas Makanan: Saiz pertukaran dan Porsi’ (22, 23) and the nutrient and composition of Malaysian foods (24). If the food consumed was not listed in all these references, at least five different sources were obtained and mean values were calculated as the weight of that particular food. The collection of the 3-day dietary diary was conducted during weekdays and weekends.

The height and weight of study subjects were measured using a calibrated SECA digital weighing machine. The body mass index (BMI) was calculated using the formula of body weight (kg) divided by square roots of height (m^2^). The WHO criteria for obesity based on the BMI guidelines (25) was a reference in this study that used the following range: underweight (BMI<18.5 Kg/m^2^), normal (BMI 18.5–24.9 Kg/m^2^), overweight (BMI 25.0–29.9 Kg/m^2^), and obese (BMI≥30 Kg/m^2^).

### Ethical approval

The project was funded by the National Institutes of Health Malaysia, and the proposal was priorly reviewed and approved by the Medical Research and Ethics Committee, Ministry of Health Malaysia. The inform consent and confidentiality was obtained from the subjects beforehand.

### Data analysis

Data analyses were conducted using SPSS for Windows version 16.0 (SPSS Inc). The data included demographic characteristics and different categories of the group, and the cooking style of the seafood consumption data. After data entry, a check was made for any discrepancies, including coding numbers, typographical errors, and so on. At the initial stage, descriptive statistics were conducted to assess data normality using a one-sample Kolmogorov–Smirnov test and/or the skewness of descriptive statistics was controlled between −1 and +1, whichever is true. Normally distributed numerical results were expressed as mean±SD, and the categorical results were expressed in percentage. However, numerical data, which were not normally distributed, were expressed as median±IQR (interquartile range). Differences between groups were assessed using a Student's *t*-test, and one-way ANOVA with equal variances was assumed using LSD, for normally distributed data. Conversely, for non-normal distributed data, the differences between groups were assessed using the Mann–Whitney *U* and Kruskal–Wallis *H* test. A *p*≤0.05 was considered statistically significant.

## Results

### Subjects characteristics

Table 1 shows the socio-demographic characteristic of the study subjects. The mean age of study subjects was 43.4±16.2 years. The Chinese had significantly (*p*<0.001) the highest mean age of 48.1±16.3 years compared with the other two ethnics. A gender proportion among subjects was about equal such that the percentages ratio was 44:55 for males and females, respectively. The body weight (kg) and height (cm) measurements for different ethnics showed significantly difference (*p*<0.001) leading toward significant differences (*p*<0.001) of the overall BMI as well. The mean BMI was at the overweight range (25.4±5.5), and this is true among Malays and Indians. However, the BMI for the Chinese is at the normal range (24.4±4.9). The mean number of household was five and most of the subjects were married (71.9%), 20.6% of them were single, and only 7.5% were either widowed or divorced. The distribution of subjects throughout the regions was highest at the northern region of Peninsular Malaysia (33%), followed by the middle region (25.2%), the east region (23.7%), and finally the southern region of the Peninsular Malaysia (18.2%). The mean education years for all ethnics were 9±3.9 years. This shows that most of the subjects completed at least lower secondary school. The Chinese, significantly (*p*=0.004), had the lowest mean education years (8.4±4.1 years) compared with other ethnics.

### Fish/seafood preferences

**Fig. 2:**
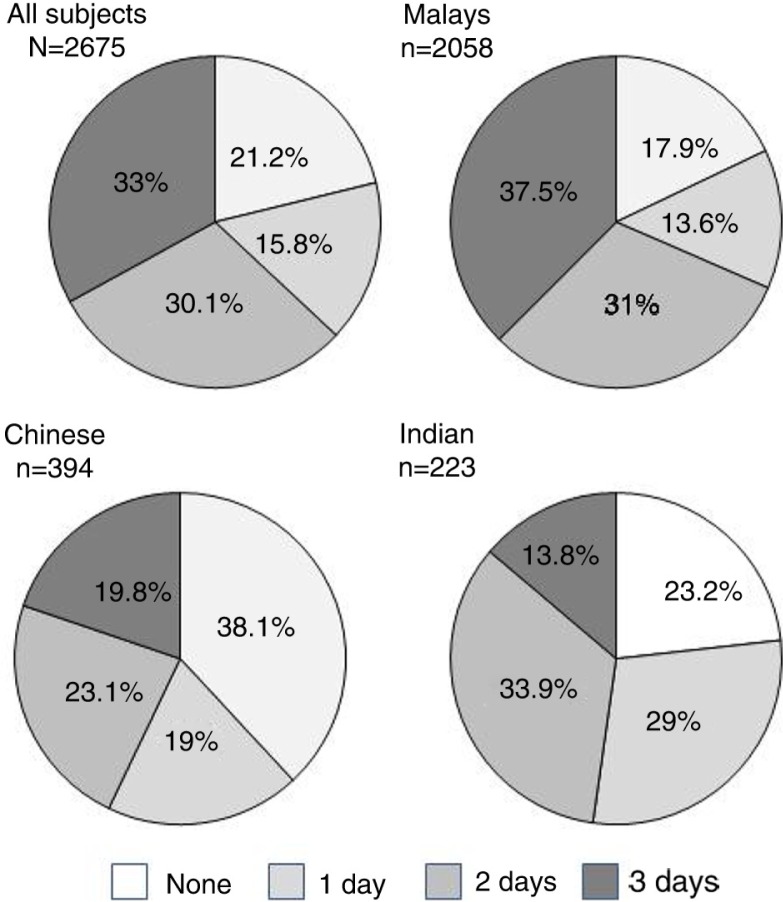
Number of days (in percentages) in which fish was consumed among the adults of different ethnicities from Peninsular Malaysia, using 3-day records of food consumption as a survey method.

Generally, the result from this study showed that 33% of the study subjects consumed fish every day from the 3 days of the food survey records (Fig. 2). Another 30.1 and 15% consumed 2 and 1 day, respectively. The remainder of 21.2% subjects did not consume any fish during the 3-day survey. Of the Malays, 38% consumed fish every day during this 3-day records compared with the Chinese (19.8%) and the Indians (13.8%). Conversely, high percentages of Chinese subjects (38.1%) did not consume any fish during the 3-day survey, followed by the Indians (23.2%) and the Malays (17.9%) (Fig. 2). The results also showed that more than half (55–57%) of these study subjects consumed seafood at least one to two meals per day during the 3-day study records.

Table 2 shows fish frequencies captured from the 3-day records of food consumption survey conducted among adults of study subjects. The most consumed fish by adults of Peninsular Malaysia was Indian mackerel. The second most consumed marine fish was anchovy, followed by yellowtail and yellow-stripe scads, tuna, sardines, torpedo scads, Indian and short-fin scads, pomfret, red snapper, and king mackerel. Other fish such as threadfin bream, croaker, marine catfish, stingray, and barramundi were also among highly preferred marine fish by study subjects. The following descending orders were another nine types of marine fish that were fairly consumed by study subjects: eel-tailed catfish, John's snapper, toli shad, big eye scads, wolf-herring, queen fish, Indian salmon, bigeye trevally, and mullet. Another 15 types of marine fish were only consumed by less than 10 times, which showed less preference by study subjects.

**Table 2:** Fish frequencies from 3-day records of food consumption survey conducted among adults of Peninsular Malaysia (*n*=2,675)

No.	Local name	English name	a*Species*	bF
Marine fish				
1	Kembung/pelaling/mabung/temenung	Indian mackerel	*Rastrelliger brachysoma, Rastrelliger faughni, Rastrelliger kanagurta*	1,397
2	Ikan bilis	Anchovy	*Encrasicholina heteroloba, E. punctifer, S. tri, Stolephorus andhraensis, S. baganensis, S. waitei, S. chinensis, S. commersonnii, S. indicus, S. dubiosus, S. insularis, Lycothrissa crocodile*	320
3	Selar kuning/pelata	Scad (yellowtail, yellowstripe, smallmouth)	*Selar crumenopthalmus, Selaroides leptolepis, Seriola dumerili, S. leptolepis, Alepes djedaba, A. melanoptera, A. vari, Atule mate, A. apercna*	280
4	Tongkol	Kawakawa/Tuna/Bonito	*Auxis thazard, Gymnosarda unicolor, Sarda orientalis, Thunnus tonggol, Euthynnus affinis, Katsuwonus pelamis, T. obesus, T. albacares*	236
5	Sardine/tamban	Sardines/pilchards	*Amblygaster sirm*, *Spratelloides delicatulus*, *Sardinella fimbriata, S. gibbosa, Dussumieria acuta, S. albella, S. brachysoma, S. jussieui, S. lemuru, S. melanura, Spratelloides gracilis, S. delicatulus*	229
6	Cencaru	Torpedo scad	*Megalaspis cordyla*	190
7	Selayang	Scad (Indian, shortfin, and mackerel)	*Decapterus akaadsi, D. macrosoma, D. maruadsi, D. russelli, D. tabl, D. macarellus, D. kurroides, D. lajang*	172
8	Bawal (hitam and putih)	Pomfret (black and silver)	*Parastromateus niger, Pampus argenteus, P. chinensis*	168
9	Merah	Red Snapper	*Lutjanus lemniscatus, L. malabaricus, L. sanguineus, L. sebae, L. argentimaculatus, L. bohar, L. erythropterus, L. bengalensis, L. boutton, L. decussate, L. dodecacantboides*	105
10	Tenggiri	King Mackerel	*Scomberomorus commerson, S. guttatus*, *S. lineolatus*	105
11	Kerisi	Threadfin bream	*Nemipterus virgatus*, *N. peronii, N. nematophorus, N. japonicus, N. bathybius, N. hexodon, N. marginatus, N. mesoprion, N. nemurus, N. thosaporni, N. nematopus, N. furcosus, N. tambuloides, N. isacanthus, N. bipunctatus, N. vitiensis, N. zysron, N. aurora, N. balinensoides*	95
12	Gelama	Croaker	*Johnius amblycephalus, J. goldmani, J. weberi, Chrysochir aureu, Nibea soldado, O. cuvieri, Panna microdon, P. anea, J. borneensis, J. coitor, Paranibea semiluctuosa, J. carouna, Pennahia macrocephalus, J. glaucus, P. pawa, J. macrorhynus, Pterotolithus lateoides, P. maculatus, N. albiflora, J. macropterus, J. trachycephalus, Aspericorvina jubata, Kathala axillaris, Daysciaena albida, Otolithoides pama, Atrobucca kyushini, Bahaba polykladiskos, Boesemania microlepis, J. heterolepis*	90
13	Mayong/seludu/duri/pelotan	Marine catfish	*Nemapteryx caelata, Plicofollis platystomus, Osteogeneiosus militaris, Arius oetik, A. maculates, N. macronotacantha, Arius jella, Picofollis tenuispinis, P. tonggo, A. venosus, N. macronotacantha, Netuma thalassina, P. tenuispinis, A. platystomus, A. subrostratus, Hemarius sona*	63
14	Pari	Stingray	*Himantura walga, H. lobistoma*, *Gymnura poecilura, H. bleekeri, Dasyatis kuhlii*	60
15	Siakap	Barramundi	*Lates calcarifer*	46
16	Sembilang	Eel-tailed catfish	*Tandanus tandanus*, *Plotosus canius*	29
17	Jenahak	John's snapper	*Lutjanus russelli, L. johnii*	29
18	Terubuk	Toli shad	*Tenualosa toli*	20
19	Lolong	Bigeye scad	*Selar crumenophthalmus*	19
20	Parang	Wolf-Herring	*Chirocentrus dorab*	19
21	Talang	Queenfish	*Scomberoides commersonnianus, S. lysan, S. tala*	19
22	Senangin	Indian Salmon	*Eleutheronema tetradactylum*	18
23	Nyok-nyok	Bigeye trevally	*Caranx sexfasciatus*	13
24	Belanak	Mullet	*Moolgarda cunnesius, Mugil cephalus, Chelon planiceps, C. macrolepis, Valamugil buchanani, C. subviridis, C. melanopterus, Moolgarda, M. seheli engeli, Ellochelon vaigiensis, V. speigleri, Liza subviridis, L. vaigiensis, L. macrolepis, Paramugil parmatus*	10
25	Gerut-gerut	Silver Grunter	*Pomadasys argenteus, P. argyreus*, *P. kaakan, P. olivaceus, P. commersonnii, P. maculatus, P. unimaculatus, P. guoraca, P. hasta, P. multimaculatum*	8
26	Kurau	Atlantic threadfin	*Polydactylus octonemus*	7
27	Kekek	Ponyfish	*Photopectoralis bindus, Equulites elongates, Leiognathus equulus, Aurigequula fasciata, E. leuciscus, Eubleekeria splendens, L. berbis, S. indicus, Secutor insidiator, Gazza minuta, L. lineolatus, Karalla daura, N. blochii, E. rivulatus, Nuchequula nuchalis, N. gerreoides, L. brevirostris, S. ruconius, G. achlamys, E. stercorarius, L. longispinis*	6
28	Selangat	Gizzard shad	*Nematalosa come, N. galatheae, N. nasus, Anodontostoma chacunda, A. thailandiae*	6
29	Putih, ebek	Diamond trevally	*Alectis indica, A. ciliaris*	5
30	Yu	Shark	*Nebrius ferrugineus, Carcharhinus leucas*	5
31	Kerapu	Grouper	*Epinephelus sexfasciatus, E. coioides, E. morrhua, Plectropomus maculatus, Grammistes sexlineatus, E. fasciatus, E. cyanopodus, E. ongus, Variola louti, E. areolatus, P. areolatus, E. malabaricus, E. coeruleopunctatus, E. corallicola, Aethaloperca rogaa, Cephalopholis miniata, C. sonnerati, Cromileptes altivelis, E. amblycephalus, E. fasciatomaculosus, E. lanceolatus, E. magniscuttis, E. polyphekadion, E. quoyanus, E. undulosus, V. albimarginata Gracila albomarginata*	5
32	Puput	Chinese herring/slender shad	*Ilisha elongata*	5
33	Sebelah	Halibut	*Psettodes erumei*, *Pseudorhombus malayanus*	4
34	Biji Nangka	Yellow goatfish	*Upeneus sulphureus*	3
35	Belut	Eel	*Congresox talabonoides*	1
36	Tongsan	Bighead carp	*Hypophthalmichthys nobilis*	1
37	Salmon	Atlantic Salmon	*Salmo salar*	1
38	Kacang-kacang	Barracuda	*Sphyraena jello*	1
39	Jolong	Halfbeaks	*Dermogenys pusilla, Hemiramphus achipelagicus, H. far, H. lutkei, N. marginatus, Hyporhamphus dussumieri, H. limbatus, H. quoyi, H. xanthopterus, Rhynchorhamphus georgi, Zenarchopterus dispar, Z. ectuntio, Z. gilli*	1
Freshwater fish				
1	Keli	Freshwater catfish	*Clarias batrachus*	77
2	Haruan	Snakehead fish	*Channa micropeltes*	31
3	Sepat	Gourami	*Trichogaster trichopterus, Trichopodus pectoralis*	19
4	Patin	Iridescent shark	Pangasius hypophthalmus	17
5	Talapia	Tilapia	*Oreochromis mossambicus*	13
6	Puyu	Climbing perch	*Anabas testudineus*	9
7	Sebarau	Hampala	*Hampala macrolepidota*	7
8	Kelah	Mahseer	Tor tambroides	6
9	Jelawat	Hoven's carp	*Leptobarbus hoevenii*	3
10	Lampam	Java barb	*Barbonymus gonionotus*, *B. schwanenfeldii*	2
11	Baung	Bagrid catfishes	*Chrysichthys nigrodigitatus*	2
12	Pacu	Pacu	*Piaractus mesopotamicus*	1
Cephalopods and mollusk				
1	Udang	Prawn and shrimp	*Penaeus monodon, P. semisulcatus, M. affinis, P. japonicus, Parapenaeopsis sculptilis, M. ensis, Metapenaeus brevicornis, M. barbata, Parapenaeospsis hardwickii, P. merguiensis, P. indicus, Parapenaeospsis hardwickii, P. latisulcatus* Kishinouye	298
2	Sotong	Squid and octopus	*Loligo uyii, Cistopus indicus, L. chinensis, L. duvauceli, L. edulis, L. sibogue, Sepia esculenta, S. phuruonis*	263

a Species names were based on fish landed and/or available at the wholesale market in Peninsular Malaysia (26–28) and at the website: www.fishbase.org/ComNames/CommonNameSearchList.

b The frequency of fish obtained from the subjects of the food consumption survey (3-day records). Of the fish consumption records, 40% did not mentioned fish name, and therefore are not included in this list.

On the contrary, out of 12 types of freshwater fish consumed during the 3-day diaries, freshwater catfish acquired the highest preference by study subjects, followed by the snakehead fish. Other freshwater fish that were fairly consumed were gourami, iridescent shark, and tilapia. Another seven types of freshwater fish (climbing perch, hampala, mahseer, hoven's carp, Java barb, bagrid catfishes, and pacu) were less consumed by study subjects.

Cephalopods and mollusk were among highly preferred seafood by Malaysian and these preferences fall within the most preferred five groups of marine seafood by Malaysian adults.

### Fish cooking style preferences

The most common cooking styles obtained from the 3-day records of food consumption survey conducted among adults of Peninsular Malaysia are shown in Table 3. Deep-fried fish is the cooking style for fish/seafood that was most preferred by study subjects. This was followed by fish cooked in thick chili gravy (*masak sambal*), fish curry, fish cooked in coconut milk with other spices and flavoring (*masak lemak*), and fish cooked in thin chili gravy and other additional spices and flavorings (*Masak asam pedas*). Other cooking styles that were also highly preferred were fish boiled with asam gelugur and lemongrass (*masak singgang*), grilled fish, and deep-fried fish cooked with soy sauce and spices (*masak kicap*). Moderately preferred fish cooking styles were boiled fish, steamed, sweet sour and *masak sos*. Less preferred cooking styles for fish were soup, *masak merah, masak taucu, masak gulai tempoyak*, and *ikan sumbat sambal*. The least preferred cooking styles were *masak tiga rasa, tomyam, masak kurma, masak rendang*, and *paprik*.

**Table 3:** Frequencies of cooking style obtained from 3-day records of the food consumption survey conducted among adults of Peninsular Malaysia (*n*=2,675)

No	Cooking styles	Cooking description	aF
1	Goreng	Deep-fried seafood marinated with salt and turmeric powder.	2,021
2	Masak sambal	Deep-fried seafood cooked in chili, shrimp paste, and tamarind paste. Thick gravy.	521
3	Masak kari	Seafood curry. Cooked with curry powder and coconut milk.	401
4	Masak lemak	Seafood boiled in coconut milk, spices, and flavorings such as lemongrass, basil leaves (*Ocimum sanctum*), turmeric leaves (*Curcuma domestic*), and asam gelugur (dried *Garcinia atroviridis*).	320
5	Masam asam pedas	Seafood cooked with chili, tamarind paste, and other spices and flavorings; kesum leaves (*Polygonum minus*) and ginger flower (*Etlingera elatior*). Thin gravy.	309
6	Masak singgang	Seafood boiled with asam gelugur (dried *Garcinia atroviridis*) and lemongrass.	158
7	Bakar	Grilled (charcoal, flat pan or oven).	148
8	Masak kicap	Deep-fried seafood cook with soy sauce and spices.	144
9	Rebus	Seafood boiled with onion, salt, and asam gelugur (dried *Garcinia atroviridis*).	67
10	Stim	Fish seasoned with soy sauce, coriander, onion, and other flavorings, cook with steamer.	45
11	Masak masam manis	Deep-fried seafood cooked in a sauce containing sugar, vinegar, or lemon and pineapple (especially of Chinese-style food).	43
12	Masak sos	Deep-fried seafood cooked with chili and tomato sauce.	40
13	Sup	Seafood boiled with soup spices and some flavorings (onion, garlic, ginger, and celery).	27
14	Masak merah	Deep-fried seafood cooked in red spicy tomato sauce other spices and flavorings.	25
15	Masak taucu	Deep-fried seafood cooked with minced bean paste, onion, and vegetables	21
16	Masak Gulai tempoyak	Seafood cooked with fermented durian, chili, and coconut milk.	19
17	Sumbat sambal	Completely dressed fish, which is stuffed with sambal belacan (chili and shrimp paste) and grated coconut before being deep-fried or grilled.	11
18	Masak tiga rasa	Deep-fried fish vinaigrette with sweet, hot and sour gravy, and mixed vegetables.	4
19	Tomyam	Seafood cooked in Thai hot and sour soup. The basic broth is made of stock and fresh ingredients such as lemongrass, kaffir lime leaves, galangal, lime juice, fish sauce, and crushed chili peppers.	2
20	Masak kurma	Seafood cooked with korma powder, coconut milk, yogurt, and other spices and flavorings.	2
21	Masak rendang	Seafood cooked with coconut milk and a paste of mixed ground spices, which includes ginger, galangal, turmeric leaves, lemon grass, garlic, shallot, chilies, and other spices.	1
22	Paprik	Deep-fried seafood cooked in chili paste, oyster sauce, mixed vegetables, spices, and flavorings.	1

a The frequency of cooking styles obtained from the subjects of the food consumption survey (3-day records).

### Fish consumption among different ethnicity

Table 4 showed the fish consumption among different ethnicities in Peninsular Malaysia. There were no significant differences (*p*>0.05) for each category of fish/seafood across different ethnics. However, the consumption of marine fish showed marginal significant differences (χ^2^=5.7; *p*=0.058), such that the Indians consumed less of this category (81±89 g/day) compared with the other two ethnic groups (Malays=100±88 g/day; Chinese=110±88 g/day). Subjects consumed more fish (marine fish 100±87 g/day; freshwater fish 106±130 g/day) compared with other types of seafood, namely cephalopod and mollusk. Although the higher frequencies of consumption of cephalopod and mollusk were observed in Table 2, the amount of consumption per person per day was much lower compared with marine fish and freshwater fish. No significant differences (*p*>0.05) among different ethnics were shown for the consumption of seafood from different habitats. However, the results showed that study subjects consumed more demersal fish (112±109 g/day) compared with fish from other habitats.

**Table 4:** Seafood consumption (g/person/day) (±IQR)^a^ among adults of different ethnics in Peninsular Malaysia

	Ethnicity				
			
Food category	Malaysian (*n*=2,058)	Chinese (*n*=394)	Indian (*n*=223)	Total (*n*=2,675)	χ^2^ (*p*)^f^
Marine fish	100±88	110±88	81±89	100±87	5.70 (0.058)
Freshwater fish	105±130	260±374	220	106±130	2.80 (0.242)
Cephalopod	90±70	101±48	80±240	90±70	0.22 (0.896)
Mollusk	41±70	30±96	30±88	40±70	0.84 (0.656)
bHabitat category					
Demersal fish	112±110	100±146	145±171	112±109	1.63 (0.443)
Others	97±81	110±83	82±86	97±80	4.55 (0.103)
cSeafood category					
Indian mackerel	80±57	80±66	110±52	80±66	5.32 (0.070)
Anchovy	22±28	30±33	30±58	24±28	3.43 (0.180)
Scad (Yellowtail, Yelowstripe, and Smallmouth)	100±74	125±127	100±117	108±74	1.04 (0.594)
Kawakawa/Tuna/Bonito,	60±55	120	60	60±55	2.78 (0.249)
Sardines, and Pilchards	43±47	229	41±62	48±56	1.54 (0.463)
Torpedo scad	110±73	100±9	–	100±73	0.08 (0.778)
Scad (Indian, shortfin, and mackerel)	90±61	315	–	92±66	1.49 (0.222)
Pomfret (Black and Silver)	91±84	95±35	60±14	90±60	7.89 (0.019)
Cooking category					
Deep fried^d^	100±79	85±66	80±72	97±77	6.57 (0.037)
Boiled^e^	101±94	110±122	80±77	99±93	7.39 (0.025)
Grilled and Steamed	81±66	68±65	89	80±59	1.99 (0.369)
Intake of seafood per meal					
Breakfast	60±50	177±154	75±117	60±51	6.67 (0.036)
Lunch	90±66	80±55	78±50	88±62	15.35 (0.000)
Dinner	93±67	89±87	85±78	91±68	2.91 (0.234)
Total consumption	175±143	152±133	136±141	168±140	16.25 (0.000)

IQR is the interquartile range.

a The calculations were based on the 3-day records from Food Consumption Survey conducted throughout Peninsular Malaysia. The portion size of seafood was based on published local data (22–24).

b Classifications were referred to the publication by the Department of Fisheries Malaysia (27). Others included pelagic, reef-associated, benthopelagic, and bathypelagic.

c The list of seafood was based on the most frequently consumed (frequent count >150) (Table 2).

d Deep-fried fish and any cooking styles that involved the deep-frying of seafood before the addition of gravy and other spices. Examples are masak sambal, masak kicap, masak masam manis, masak sos, masak merah, masak taucu, sumbat sambal, masak tiga rasa and paprik (Table 3).

e Seafood cooked with various other ingredients, such as spices and flavorings and often containing clear or thick liquid/gravy, with onion, garlic, or other spices stir-fried. Examples are masak kari, masak lemak, masak asam pedas, masak singgang, rebus, sup, masak gulai tempoyak, tomyam, masak kurma, and masak rendang (Table 3).

f Significant differences (*p*<0.05) between different ethnics were evaluated using the Kruskal–Wallis H test.

The Indians consumed more Indian mackerels compared with the other ethnicities, as shown by marginal significant differences (χ^2^=5.32; *p*=0.070) between different ethnic groups. On the contrary, the Malays and Chinese consumed significantly (χ^2^=7.89; *p*=0.019) higher amounts of black and silver pomfret compared with the Indians. None of the consumption patterns for the other types of fish showed significant differences (*p*>0.05) between ethnicities. However, the total consumption of different seafood categories indicated that the subjects consumed more yellow tail and yellow-stripe scads (108±74 g/day) and torpedo scads (100±73 g/day) compared with other fish/seafood categories. Details on the consumption data disclosed that none of the Indians consumed torpedo scads, Indian scads, and short-fin scads within the 3-day survey. Only one Chinese subject consumed tuna, sardines, Indian, and short-fin scads captured during this study.

There were significant differences between consumption of deep-fried fish (χ^2^=6.57; *p*=0.037) and boiled fish (χ^2^=7.39; *p*=0.025) among different ethnicities. The Malaysians preferred (*p*=0.037) deep-fried fish, while the Chinese were in favor (*p*=0.025) of boiled fish. The total consumption of each cooking category showed approximately similar amounts for the different ethnicities, with grilled and steamed fish consumed least.

The Chinese consumed significantly (*p*=0.036) more seafood during breakfast, while the Malays consumed more during lunchtime (*p*<0.001). No significant differences were shown for the consumption of seafood in dinner meals (*p*>0.05) by different ethnics. The results showed that more seafood were consumed during dinner (91 g/day) compared with lunchtime (88 g/day), but the least was consumed during breakfast (60 g/day).

Total consumption of seafood by adult of different ethnicity from Peninsular Malaysia was 168±140 g/day. The results from this study showed that the amount of seafood consumed by different ethnics were significantly different (*p*<0.001). The Malays consumed the highest amount (175±143 g/person/day), followed by the Chinese (152±133 g/person/day) and the least amount by the Indians (136±141 g/person/day).

### Fish consumption by different factors

Table 5 showed the differences of seafood consumption by the adults of Peninsular Malaysia with different factors. Amount of seafood consumed showed significant difference (*p*<0.01) between age groups. The results showed that older people consumed significantly more fish (*p*=0.02) compared with the younger ones. No significant differences (*p*>0.05) were shown between seafood consumption and different gender, as well as BMI status. Married subjects consumed significantly more seafood (*p*<0.001) compared with those who were single, widowed, or divorced. Subjects resided around the northern and eastern coastal regions, consumed significantly (*p*<0.001) more seafood compared with other areas. Subjects with higher years of education consumed significantly less seafood (*p*<0.001), compared with the other groups with less educational years.

**Table 5:** Comparison of seafood consumptions in Peninsular Malaysia with different factors (*n*=2,675)

No	Factors	*n*	Median±IQR	χ^2^ (*p*)
1	Ethnic			
	Malay	1,694	175±143	^KW^16.253 (0.000)
	Chinese	244	153±134	
	Indian	172	138±139	
2	Age			
	18–40 years old	905	160±141	^KW^12.17 (0.002)
	41–59 years old	859	174±141	
	≥60 years old	368	176±139	
3	Gender			
	Male	932	174±140	^MW^538955 (0.180)
	Female	1,197	164±142	
4	BMI			
	Normal	881	172±140	^MW^365744 (0.588)
	Others	843	167±144	
5	Marital status			
	Married	1,568	176±142	^MW^361617 (0.000)
	Others	539	148±133	
6	Residential area by state			
	North (Perlis, P. Pinang, Kedah, and Perak)	742	186±145	^KW^75.903 (0.000)
	Middle (Selangor, WP Kuala Lumpur, and N. Sembilan)	527	147±126	
	South (Melaka and Johor)	325	154±119	
	East (Pahang, Terengganu, and Kelantan)	538	182±155	
7	Years of Education			
	≤6years	636	172±133	^KW^19.436 (0.000)
	7–11 years	965	170±142	
	≥12 years	299	136±135	

^KW^The Kruskal–Wallis and ^MW^Mann–Whitney U tests were applied.

## Discussion

The present study explored a cross-cultural difference for patterns of fish/seafood consumption among adults of the three major ethnicities in Peninsular Malaysia. This study had several advantages whereby fish/seafood consumption patterns were collected using successive 3-day record surveys (dietary diaries) throughout both rural and urban areas of Peninsular Malaysia. This study also involved all adults from selected households and covered both the weekdays and weekends. A good cooperation obtained from study subjects resulted in high response rates (89.2%) of the survey demonstrating obliging Malaysians, a supportive and mature society.

Malaysia is a multiracial and multi-religion community in which all populations accept fish and seafood compared with meat-based protein foods, such as pork and beef, which are prohibited among Muslims and Hindus, respectively. Malaysians consume fish and seafood not only because of these concerns but also because it is the cheapest meat protein available in this country (29). This is because a long coastal line of 4,800 km facing both the South China Sea and the Straits of Malacca covers Peninsular Malaysia, where coastal and deep-sea fisheries activities are prime sectors (30). Results from this study verified these scenarios by showing that many Malaysians (33%)—especially the Malays—consumed fish/seafood every day or at least 4 to 5 days a week (30%). Another group (15%) consumed fish and seafood twice a week. These results, in summary, show that about 78% of Malaysians consumed fish at least twice per week. In addition, the results also showed that many subjects consumed seafood every day or at least twice a day. These findings are summarized in Fig. 2. Findings from our study were in agreement with another study (31) that reported that many of the study subjects, farmers in Muda Irrigation Area in Kedah, consumed fish (22.7%) and other seafood (22.2%) one to three times per week. Similar findings were also reported in Singapore, another South East Asian country, where people consumed fish and seafood every day and sometimes twice a day. In addition, people consumed more than one meat or fish and seafood for most of their meals per day (1).

The frequent intake of fish and seafood could also be explained by way of the traditional eating cultures practiced in this country and countries with similar eating pattern, especially around Southeast Asia. It is common for Malaysians to eat at least four times a day (breakfast, lunch, tea, and dinner) and the multiracial Malaysians consumed cooked rice or ‘nasi putih’ with side dishes of variable cooking styles of the fish and seafood, meat or chicken, and vegetables twice a day (32). These foods were served, especially during lunch and dinner. Meanwhile, most Malaysians enjoyed ‘nasi lemak’ (rice cooked in coconut milk), fried rice, fried noodle, and ‘roti canai’ (circular flatbread) for breakfast. These dishes were served either together with a variety of cooking styles of meat-based protein or added as an ingredient in the main dishes. For example, ‘nasi lemak’ is usually consumed with anchovy or other seafood, such as prawn, squid, or cockles cooked in chilies. Fish/seafood was also used as an ingredient in many other Malaysian traditional snacks/kuih (cakes), such as prawn fritters, spring rolls, curry puff, ‘cucur badak’ (deep-fried round mashed sweet potato with wheat flour and prawn and coconut fillings), ‘pulut udang’ (glutinous rice cooked with coconut milk and prawn and coconut fillings), prawn/fish crackers, and the immensely popular ‘keropok lekor’ (deep-fried fish sausages), which are popular dishes during tea.

A nation-wide study, the Malaysian Adults Nutrition Survey (MANS), carried out between October 2002 and December 2003, revealed that an average of two and a half plates of cooked rice was consumed by 97% of population twice daily with one medium marine fish per day. Examples of preferred marine fish consumed in this study were ‘ikan kembung’ (Indian mackerel), ‘ikan tenggiri’ (Spanish mackerel), and ‘ikan merah’ (red snapper) (2). The findings from this current study were also in agreement with this nation-wide study in which ‘ikan kembung’ that mainly included three species caught in this country, *Rastrelliger brachysoma*, *Rastrelliger faughni*, and *Rastrelliger kanagurta*, were among the most preferred fish by the study subjects, while Spanish mackerel and red snapper were among 10 most preferred fish. Another important finding from the nation-wide study was that the daily intake of marine fish, including anchovy, was significantly higher among rural adults (51%) compared with urban adults (34%) (2). On the contrary, in this current study, anchovy was the second most preferred fish by study subjects, notwithstanding its mean consumption per day (24±28 g/person/day), which was not significantly different among ethnicities. This pattern was similar to other types of fish as well, except for black and silver pomfret, for which the consumption was significantly less by the Indians when compared with the other two ethnicities.

A study on food intake conducted among the young generation in Malaysia (aged less than 30 years) revealed that Malays and others ate rice more frequently than the Chinese and Indians (33). On the intake of side dishes, the preferences went to chicken, followed by fish but among different ethnicities, the Chinese preferred chicken and meat, the Indians like shrimp and squid, while the Malays like to take fish (33). These results contradicted the findings from our study that Malays and the Chinese consumed more marine fish compared with Indians, and the differences were at the borderline of statistical significance (*p*=0.058). There were no significant ethnic differences for the consumption of cephalopods, mollusks, and freshwater fish as well. The differences may be due to an age factor because our study subjects’ ages ranged from 18 to 103 years, with an overall average of 43.4±16.2 years old. Only 40% of subjects in our study were younger than 30 years old.

Data from the study of the household consumption, by purpose, during the period of 2000–2009, showed that expenditures on food and non-alcoholic beverages accounted for about 23% of the total household expenditure for Malaysians, the largest single component (34). Another study on the share of food and fish expenditures, out of the total budget of Asian households, concluded that, overall, fish contributed the third largest share, ranging between 5 and 21% (35). The same researchers also found that fish expenditures by Malaysians were the highest (21%) followed by Bangladeshis (20%) and Vietnamese (19%). Their results highlighted the important role of fish in the animal-protein intake of most Asian households (35). In addition, economic development has shifted the Malaysian food consumption trend away from basic staple food (i.e. rice) and other traditional food crops (sweet potatoes, cassava, pulses, and oil seeds) to non-traditional, staple, wheat-based items, and meat, fruit and vegetables (36–39). Income, actual prices, relative prices, and demographic factors (38) are the driving forces behind the changes in the Malaysian food consumption trend. Of the widening role of income growth, food demand has shifted toward high-quality-differentiated characteristics, such as freshness, safety, texture, and appearance (38). Moreover, consumers were more willing to pay for these qualitative characteristics (38). In affluent societies like Malaysia, the diversification in the food basket is more likely to be filled by more non-staple foods for the calorie intake, hence implying a stronger purchasing power of the society to demand for higher value food products mainly (meat and fish) and functional food products (mainly vegetable and fruits) (38). A study by Sheng and co-workers had estimated expenditure elasticity among Malaysians and reported that the demand for meat (1.4064), fish (1.244), vegetables (1.1729), and fruits (1.0905) are likely to grow faster than other traditional main energy sources, such as rice (0.9091) and bread and other cereals (0.3177), corresponding to the positive income effect (40). Malaysians with higher income level will seek for a better food quality in terms of nutrition, hygiene, organic, and other features. According to another study by the same group of researchers (41), Malay consumers have different food consumption patterns than the general Malaysian diets. According to them, the Malay consumers increased their consumption of rice more than higher value food (meat and fish) and functional foods (vegetable and fruits). Their findings also showed that Malay consumers are sensitive to the prices of most food products, especially rice (41).

As the Malaysian lifestyle shifts from rural to urban, diet and activity levels have changed accordingly, such that large numbers of the urban population habitually eat out (42). Eating habits have shifted to the convenience of prepared and processed meals, hence changing the food consumption pattern of Malaysian communities. Traditional diets are being replaced with diets higher in fats, salts, and animal products and often with a lower intake of fresh fruits and vegetables (37). The change from traditional to fast-food eating habits is a major factor in the rising epidemic of obesity and associated chronic diseases in this country (43). Nevertheless, meals prepared at home are still favored, especially by those in rural areas, where older Malaysians still preferred traditional meals (32, 42). The younger generation seeks convenience options, such as noodles, fried rice, and soup (32). It was reported that the practice of eating out had become a trend among urban workers, students, and even families because they could not go home to eat (42) or because there was nobody at home to prepare home-cooked meals (44). Factors such as working away from home, working mothers, and food varieties served in many premises encouraged the practice of eating out (42, 44). In this country, strong players in food services include the mamak and kopitiam restaurants, where one can enjoy traditional food such as beef rendang (dry spice coconut beef dish), Laksa (tangy fish noodle soup), char kway teow, fried noodle, roti canai, and so on (32). Food caterers were also available to serve at formal functions such as meetings, seminars, or religious and family occasions (42). This pattern of eating changes, however, may not affect the pattern of fish and seafood consumption among elder people but may affect the younger generation in Malaysia.

The trend of the per capita consumption of fish is consistent with the increase in national wealth, which shifted almost 47% in 2000, as compared with 1985 (43). The consumption of fish per person in Malaysia, in 2005, was around 57.3 kg/person/year (39). These data were equivalent to data from our study, in which the overall fish consumption by Malaysian adults was 168±140 g/person/day or 54.77 kg/person/year. With this level of fish consumption, Malaysians ranked third after the Japanese (64.7 kg/person/year) and Koreans (51 kg/person/year) (39). Compared with other regional countries, the subjects in this study consumed more fish and seafood than those in Thailand (31 kg/person/year), Philippines (29.6 kg/person/year), The Republic of China (25.4 kg/person/year), Indonesia (19 kg/person/year), Bangladesh (10.9 Kg/person/year), and India (4.6 kg/person/year) (36, 39). Total consumption of fish and seafood in our study was around 40% higher when compared with Western countries such as Australia (22.7 kg/person) and the United States (23.4 kg/person) (39). The fish consumption by Spanish (range: 50.9–72 g/person/day or 18.6–26 kg/person/year) and the Norwegians (range: 42.9–63.3 g/person/day or 15.7–23.1 kg/person/year) (45) were equivalent to the intake by Australia and the United States as reported by Warr and co-workers (39). However, for other European countries, fish consumption was even lower, ranging from 4 to 23 kg/person/year or 13–72 g/person/day (45).

York and Gossard had studied the influenced of fish consumption by cultural or geographical regions and summarized that consumption trends cannot be explained by economic or ecological perspectives alone (3). They reported that for each $1,000 of per capita GDP, Asians eat 2.31 additional kilograms per year of fish, whereas Westerners eat only 0.79 additional kilograms (3). It appears that economic development spurs Asians to eat considerably more compared with other cultural regions and it is similar for non-Asian regions in the consumption of meat (3). In both Asia and Europe, the low proportion of freshwater fish in per capita fish consumption indicated the preference for marine fish (inclusive brackish-water fish). This trend is quite evident in areas where aquaculture is growing fast, such as East and Southeast Asia. Moreover, the majority of the cultured marine species are high value and depend on high-quality complete diets (46). Factors influencing fish consumption included an increment in income and urbanization among populations in developing countries, while increased consumer awareness of the health and nutritional benefits of seafood, standardization, and the availability of products and cheaper prices affected consumption in developed countries (46). The relationships between income and urbanization, and fish consumption are clearly important factors to be taken into consideration in the calculation of future fish demand and the type of fish because of the global trend toward urbanization (46).

Medical research had shown that substituting the intake of meat with a food mix in which fat fish or lean fish and fish oil, combined with vegetables, may improve the quality of fat consumption, reduce consumer's calorie intake, and prevent lifestyle diseases (47). A study of the importance of seafood as a nutrient source in the diet of Belgian adolescents concluded that increased seafood consumption would lead to a higher intake of EPA, DHA, and vitamin D. These long-chain omega-3 PUFAs can be associated with several health benefits, such as a reduction in the risk of coronary heart disease, a decrease in mild hypertension, prevention of certain cardiac arrhythmias, and sudden death (48). In line with these researches, Verbeke and Vackier had applied the theory of planned behavior for understanding the determinants of fish consumption behavior among 429 Belgians in 2003 (11). They reported that the taste and healthy image of fish are two well-appreciated characteristics, but the bones in fish and the price of fish are identified as the most likely attitudinal barriers to more frequent fish consumption. Another study conducted among Norwegian women, aged 45–69 years, addressing health and seafood consumption had revealed that seafood consumption increased with increasing belief in the idea that diet is important for health, the use of medicine for cardiovascular disease, other healthy eating habits, increasing age, increasing household size, decreasing family income, and residence in coastal areas (18, 47).

Consumption of fish and seafood can either be essential for a healthy and well-balanced diet or may pose a health risk to consumers from environmental toxicants. Many developed countries had evaluated and issued fish advisories or bans to limit the exposure to contaminants that may accumulate in fish tissue. Information on fish consumption rates is necessary in order to accurately assess exposure to harmful chemicals from the ingestion of contaminated fish (4). Therefore, the main purpose of the study in collecting these fish and seafood data is to calculate and assess the health risk of exposure to contaminants from fish consumption, which will be reported in the next article. The variables from these data were analyzed in detail in order to correctly reflect consumption rates by particular ethnicities, different age groups, socioeconomic statuses, types of fish/seafood, and sources of fish and seafood.

## Conclusions

The present results were obtained by applying a successive 3-day food record survey throughout both rural and urban areas and were conducted among subjects aged 10 or more years, selected from the three major ethnic groups in Peninsular Malaysia. Many other analyses would be possible because of the large set of recorded variables. For example, a subsample of 10- to −17-year-old children and teenagers had already been analyzed with regard to food consumption patterns and obesity (49). In this paper, the main results illustrated the most relevant aspect of fish and seafood consumption patterns for the adult population. The discussion has emphasized fish consumption frequencies or most consumed fish and seafood, most preferred cooking style, the amount of fish and seafood consumed by different types and groups of fish and seafood, cooking style, and the intake of fish and seafood per meal by three major ethnicities in Malaysia.

The advantage of the study is that the results may generalize the consumption pattern of fish and seafood among Malaysian adults as all adults in a selected household unit was included as study subjects. Moreover, the applied prospective diet record implemented in this study may offer higher accuracy of fish and seafood consumption estimates. Similar methodology may be applied for future research.

The limitation of this study was the poor response to the type of fish consumed because 40% of the fish and seafood consumption records do not mention the fish by name. Therefore, the calculation for fish and seafood consumption data were only included in the total seafood consumption. This data deficiency might be due to limited knowledge and the inability of the study subjects to identify fish type or name. Despite its limitations, this study provided a necessary update of information on fish and seafood consumption in Malaysia. Although a few similar food consumption surveys have been conducted at the national level by other researchers, our study complements those studies by providing detailed information on fish and seafood consumption. The data collected is beneficial for the purpose of health risk assessment of the intake of contaminants through fish and seafood consumption. Lastly, the experience acquired while conducting this study will be very useful for refining survey tools and data-processing procedures for future studies.
